# Supplementary material for: Health Literacy Environment of Breast and Cervical Cancer among Black African Women Globally: A Systematic Review Protocol of Mixed Methods
Source: Int J Environ Res Public Health. 2020 May 1;17(9):3158. doi: 10.3390/ijerph17093158 (PMC7246761; doi:10.3390/ijerph17093158)
Supplement: Supplementary file 1 [file ijerph-17-03158-s001.zip › ijerph-762432-supplementary.docx]

**Supplimentary files**

**Table S1: Full search strategy**

***PubMed searching strategy***

| 1 | (“health literacy” or “health information” or “health awareness” or “health knowledge” or numeracy or literacy or education or educat* or learning) |
| --- | --- |
| 2 | (“breast cancer” or “breast ca” or “cervical cancer” or “cervical ca” or “cancer screening” or cancer or ca or “pap smear”) |
| 3 | (woman or women or girl or “reproductive age”) |
| 4 | (“African refugee” or Black or “African immigrant” or “African migrant” or “people of color” or settler) |
| 5 | 1 and 2 and 3 and 4 |

***Web of Science searching strategy***

| 1 | TS= (“health literacy” or “health information” or “health awareness” or “health knowledge” or numeracy or literacy or education or educat* or learning) |
| --- | --- |
| 2 | TS= (“breast cancer” or “breast ca” or “cervical cancer” or “cervical ca” or “cancer screening” or cancer or ca or “pap smear”) |
| 3 | TS= (woman or women or girl or “reproductive age”) |
| 4 | TS= (“African refugee” or Black or “African immigrant” or “African migrant” or “people of color” or settler) |

***Scopus searching strategy***

| 1 | ALL (“health literacy” OR “health information” OR “health awareness” OR “health knowledge” OR numeracy OR literacy OR education OR educat* OR learning) |
| --- | --- |
| 2 | ALL (“breast cancer” OR “breast ca” OR “cervical cancer” OR “cervical ca” OR “cancer screening” OR cancer OR ca OR “pap smear”) |
| 3 | ALL (woman OR women OR girl OR “reproductive age”) |
| 4 | ALL (“African refugee” OR Black OR “African immigrant” OR “African migrant” OR “people of color” OR settler) |

***CINAHL Searching strategy***

| S1 | Tx “health literacy” or “health information” or “health awareness” or “health knowledge” or numeracy or literacy or education or educat* or learning |
| --- | --- |
| S2 | Tx “breast cancer” or “breast ca” or “cervical cancer” or “cervical ca” or “cancer screening” or cancer or ca or “pap smear” |
| S3 | Tx woman or women or girl or “reproductive age” |
| S4 | Tx “African refugee” or Black or “African immigrant” or “African migrant” or “people of color” or settler |

**Table S2: JBI quality appraisal and selection tool**


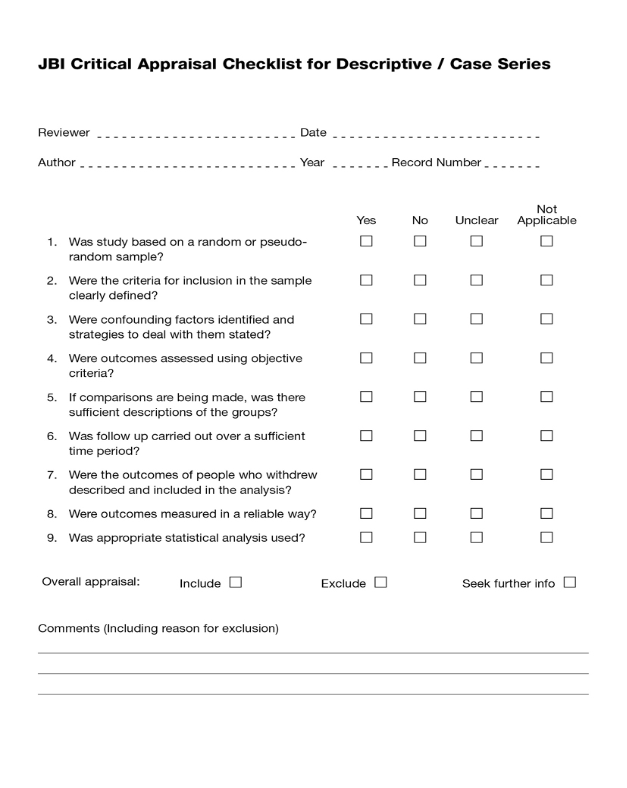


**Insert page br****this is a test message**


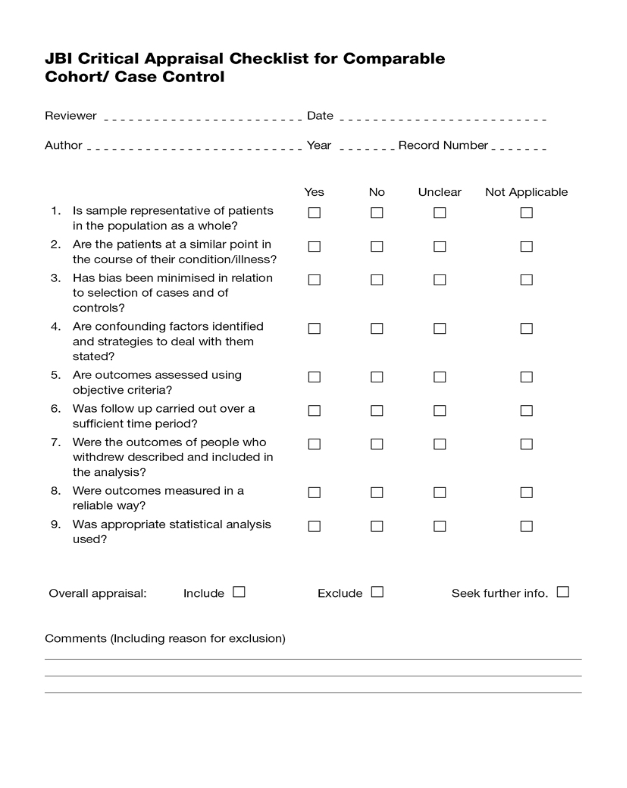


**Insert page break**

**
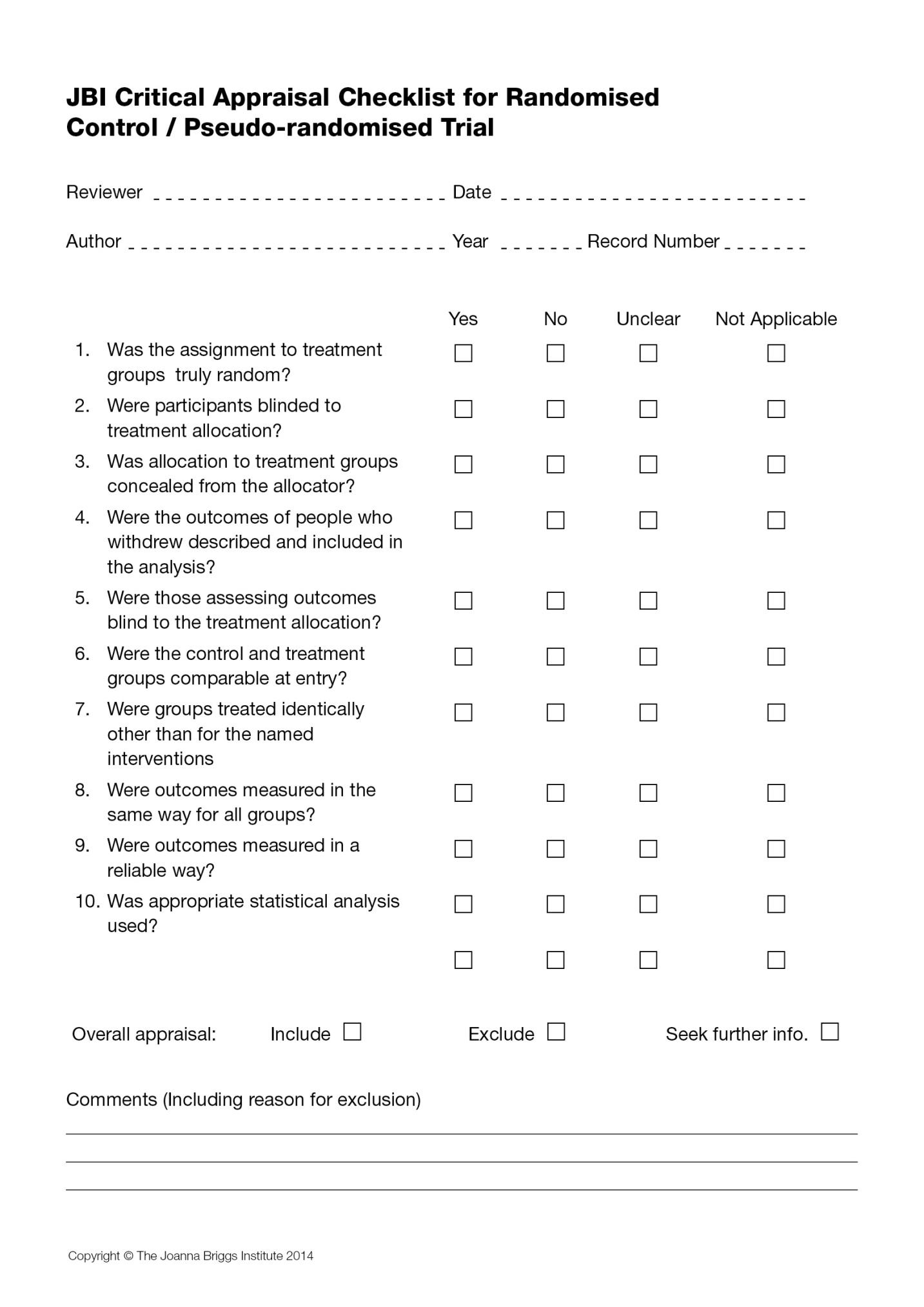
**

**
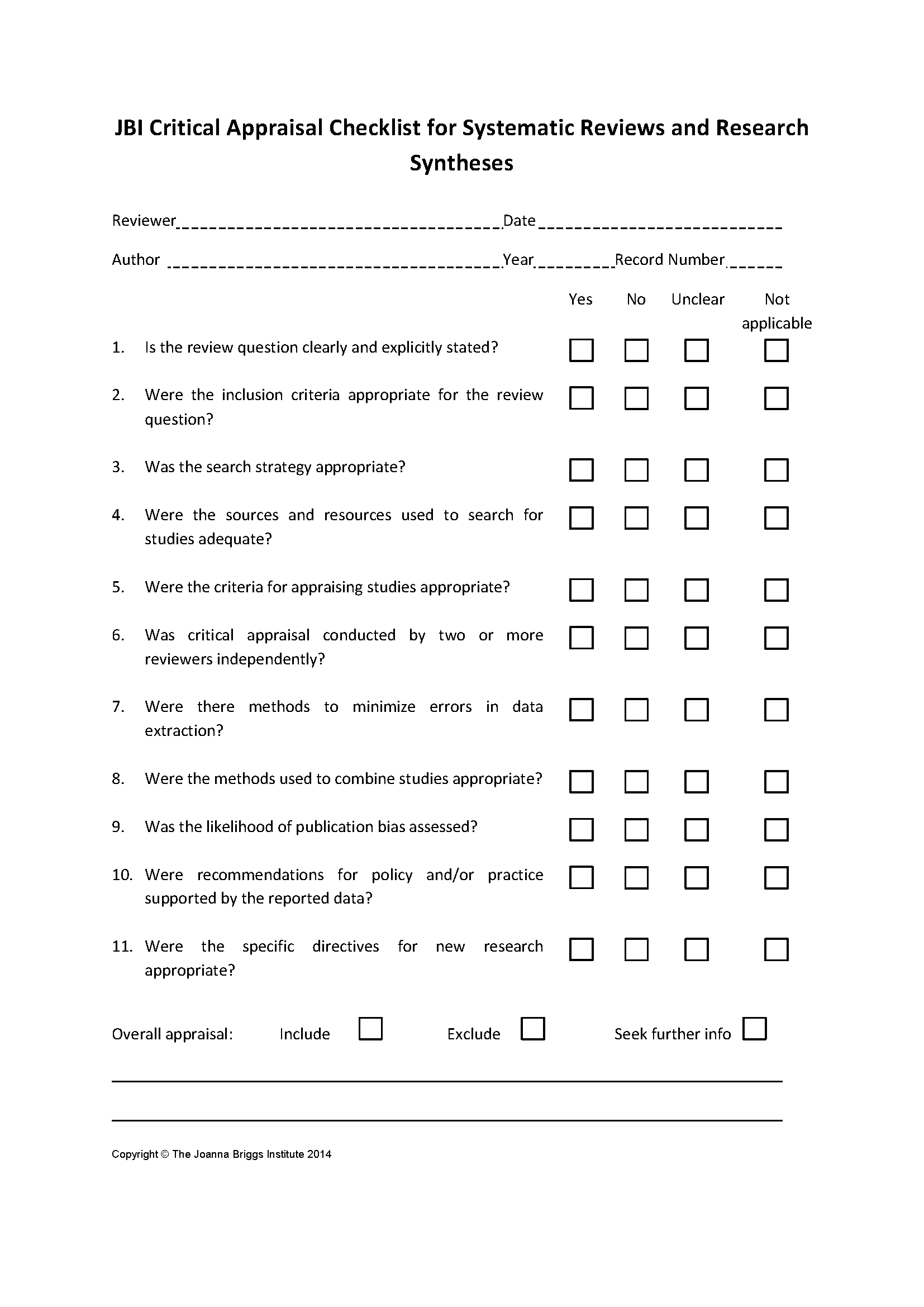
**

**
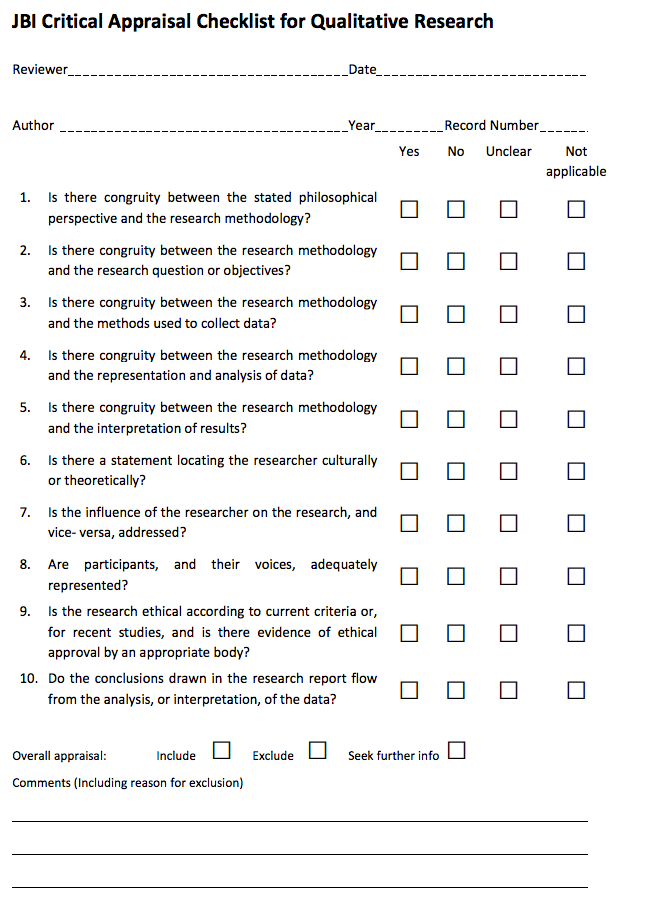
**

**Table S3: JBI-data extraction instruments**


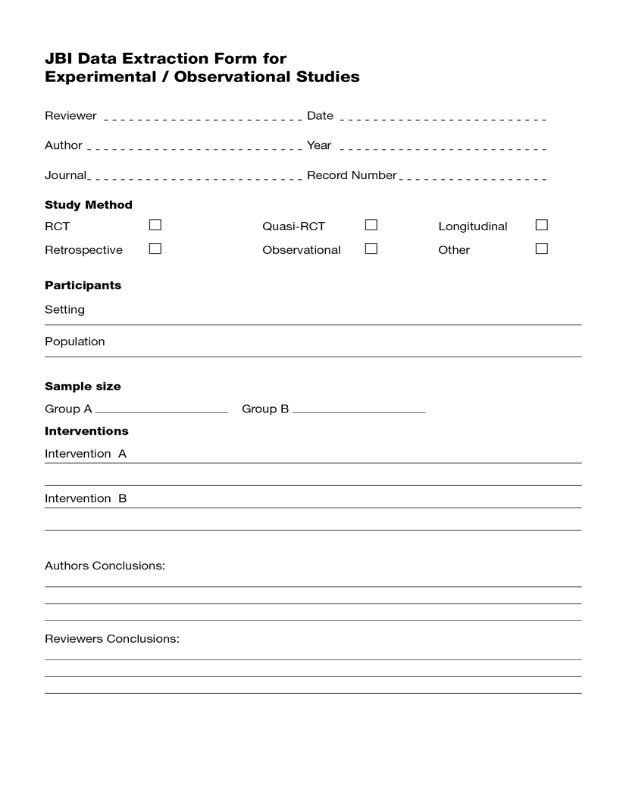


**
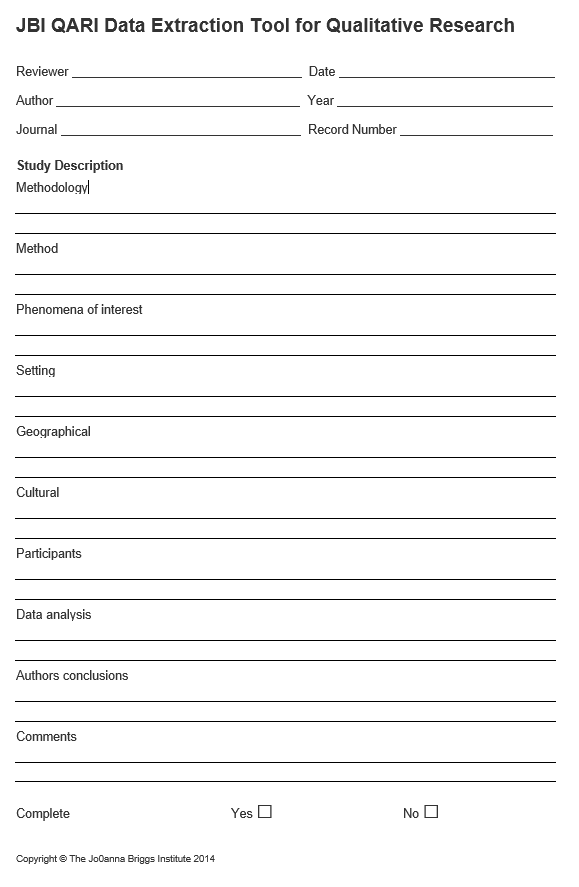
**
